# Cognitive and Neuroanatomic Accounts of Referential Communication in Focal Dementia

**DOI:** 10.1523/ENEURO.0488-18.2019

**Published:** 2019-09-26

**Authors:** Meghan Healey, Nicola Spotorno, Christopher Olm, David J. Irwin, Murray Grossman

**Affiliations:** 1 Penn Frontotemporal Degeneration Center and Department of Neurology, University of Pennsylvania Perelman School of Medicine, Philadelphia, Pennsylvania 19104; 2 Neuroscience Graduate Group, University of Pennsylvania, Philadelphia, Pennsylvania 19104; 3 Clinical Memory Research Unit, Malmo, Department of Clinical Sciences, Lund University, 221 00 Lund, Sweden; 4 Department of Radiology, Penn Image Computing and Science Laboratory, University of Pennsylvania Perelman School of Medicine, Philadelphia, Pennsylvania 19104; 5 Department of Pathology and Laboratory Medicine, Center for Neurodegenerative Disease Research, University of Pennsylvania Perelman School of Medicine, Philadelphia, PA 19104, USA

**Keywords:** frontotemporal degeneration, language, perspective taking, prefrontal cortex, referential communication, social cognition

## Abstract

The primary function of language is to communicate—that is, to make individuals reach a state of mutual understanding about a particular thought or idea. Accordingly, daily communication is truly a task of social coordination. Indeed, successful interactions require individuals to (1) track and adopt a partner’s perspective and (2) continuously shift between the numerous elements relevant to the exchange. Here, we use a referential communication task to study the contributions of perspective taking and executive function to effective communication in nonaphasic human patients with behavioral variant frontotemporal dementia (bvFTD). Similar to previous work, the task was to identify a target object, embedded among an array of competitors, for an interlocutor. Results indicate that bvFTD patients are impaired relative to control subjects in selecting the optimal, precise response. Neuropsychological testing related this performance to mental set shifting, but not to working memory or inhibition. Follow-up analyses indicated that some bvFTD patients perform equally well as control subjects, while a second, clinically matched patient group performs significantly worse. Importantly, the neuropsychological profiles of these subgroups differed only in set shifting. Finally, structural MRI analyses related patient impairment to gray matter disease in orbitofrontal, medial prefrontal, and dorsolateral prefrontal cortex, all regions previously implicated in social cognition and overlapping those related to set shifting. Complementary white matter analyses implicated uncinate fasciculus, which carries projections between orbitofrontal and temporal cortices. Together, these findings demonstrate that impaired referential communication in bvFTD is cognitively related to set shifting, and anatomically related to a social-executive network including prefrontal cortices and uncinate fasciculus.

## Significance Statement

While traditional models of language processing focus on single word and sentence comprehension, successful communication during conversational exchanges may involve additional executive resources and social perspective taking. Here, we report a novel study of non-aphasic patients with behavioral variant frontotemporal dementia (bvFTD) who have documented deficits in social and executive function but relatively preserved language. Our findings demonstrate that patients with bvFTD have difficulty coordinating perspectives with a conversational partner in a referential communication task. Patient impairment was related to disease in a network of prefrontal regions associated with social functioning and mental set shifting, highlighting the essential contribution of non-language brain regions to daily communication.

## Introduction

Language does not exist as some arbitrary, surface phenomenon, but rather serves a critical function: to communicate. Indeed, as much as 70% of our waking time is spent in some form of communication (Klemmer and Snyder, 1972): we chat on the phone with friends, give directions to strangers, and make presentations at work. When language is used in these contexts, functioning to communicate something to someone, it is inherently a task of social coordination (Clark, 2011, 2012). Indeed, for an interaction to be successful, speakers and addressees must establish shared mental representations and mutual understanding with one another. A canonical example of this is seen in referential communication, when a speaker must select the attributes of an object or referent in such a way that allows the address to identify that referent (Bowman, 1984). Accordingly, referential communication requires an individual to (1) track and adopt a conversational partner’s perspective and (2) maintain and shift between the numerous elements relevant to the ongoing exchange. In this sense, language use is strategic—it is both socially and executively demanding. Therefore, we ask the following: how do social and executive processes contribute to daily communication skills?

Surprisingly little is known about the cognitive and neural mechanisms of referential communication. Early examinations of language neurobiology focused on two hubs in left peri-Sylvian cortex, including inferior frontal gyrus (IFG; “Broca’s Area”) and posterior superior temporal gyrus (“Wernicke’s Area”). This seminal view, however, was based primarily on studies examining single-word and sentence processing and largely ignored how language operates in context. More recently, theoretical and technical developments have made it possible to investigate the neural basis of discourse processing—that is, the social use of language. For example, Xu et al. (2005) used fMRI to compare the neural correlates of words, sentences, and narratives. The authors found that peri-Sylvian regions are active regardless of context, but regions outside of the core language network, including medial prefrontal cortex (mPFC), temporoparietal junction, and precuneus, are only engaged for narratives. Few studies have examined the neural basis of referential communication per se, but one recent fMRI experiment manipulating common versus privileged ground information also suggests the mPFC comes on-line when language production necessitates speaker perspective taking (Vanlangendonck et al., 2018). Similar findings have been reported in other narrative-based studies (Troiani et al., 2008; Saur et al., 2010; AbdulSabur et al., 2014). Here, we test this hypothesis that nonlanguage brain regions in prefrontal cortex are critical to referential communication, using a lesion-model approach and a carefully controlled experimental task.

While fMRI studies in healthy adults can associate patterns of neural activity with ongoing behavior, it is a correlative technique that cannot identify which brain regions are truly necessary for a given task. Therefore, it is important to complement fMRI studies with converging evidence from patient studies. Behavioral variant frontotemporal degeneration (bvFTD) is a young-onset neurodegenerative disease characterized by social and executive limitations due to progressive atrophy in frontal and anterior temporal cortices (Rascovsky et al., 2011). Importantly, despite disease in anatomically relevant areas, linguistic ability is relatively preserved in bvFTD, which makes the group an ideal lesion-model for studies examining social and executive components of language (Kumfor et al., 2017). Previous work using this same logic has also examined social discourse and referential communication in patients with bvFTD (Rankin et al., 2009; McMillan et al., 2012; Shany-Ur et al., 2012; Gola et al., 2015; Healey et al., 2015) In one such study (Healey et al., 2015), patients had to generate brief speech samples describing the movement of a target object to a conversational partner. Conditions manipulated perspective-taking demand (i.e., the amount and type of information available to both interlocutors) and executive demand (i.e., the number of competing objects in the array). Textual analyses indicated that patients with bvFTD produce descriptions that lack critical, distinguishing adjectives—a speech pattern that would drive poor communication outcomes in a real-world setting. The observed impairment was further associated with disease in medial, dorsolateral, and orbitofrontal cortices, all regions associated with a social network thought to be compromised in bvFTD (Ibañez and Manes, 2012).

We build upon this previous work in four important ways. First, past research has suggested that patients with bvFTD may be overwhelmed by the cognitive demands associated with continuous speech production, often showing a phenotype characterized by reduced rate, decreased information content, and abnormal prosody (Ash et al., 2006; Nevler et al., 2017; Vogel et al., 2017). Therefore, to isolate the social and executive components of referential communication from the underlying speech and motor components, we use a simple, forced-choice task in the present work.

Second, much of the existing work on referential communication has relied on a contrast between common ground information (mutually accessible to both interlocutors) and privileged ground information (accessible to only a single interlocutor; Brown-Schmidt et al., 2008; Heller et al., 2008; Wardlow et al., 2014). The privileged ground condition traditionally uses stimuli that physically obstruct one partner’s view and, as such, places high demands on visuospatial processing. Here, we contrast a novel “color-blind” condition with a visually identical “sighted condition” to minimize experimental confounds and target social (rather than visual) perspective taking.

Next, while deficits in executive function (EF) and working memory have been consistently demonstrated in bvFTD (Kramer et al., 2003; Libon et al., 2007), the relationship of these constructs with patients’ perspective taking remains unclear (Bertoux et al., 2016). For example, while some work demonstrates that EF and theory-of-mind are closely related in bvFTD (Snowden et al., 2003), other work demonstrates that the two are independent and dissociable (Lough et al., 2006). To address this ongoing controversy, we adopt the widely accepted tripartite model of EFs described by Miyake et al. (2000), and separately probe the following three postulated subdomains: mental set shifting, information updating, and inhibition.

Finally, previous work, both in healthy and clinical populations, has focused primarily on gray matter (GM) contributions to language processing. However, white matter (WM) tracts also play a critical role in network activity by transmitting electrical signals across spatially separate brain regions. Therefore, even when GM regions are intact, synchronized network activity may be disrupted if there is significant WM damage. Accordingly, and because bvFTD is known to show significant WM disease (Agosta et al., 2012), we collect high-resolution diffusion tensor (DT) imaging. While the arcuate fasciculus (connecting Broca’s and Wernicke’s Area) is the primary language-associated fiber pathway (Dick and Tremblay, 2012), we predict that additional tracts, including the uncinate fasciculus (connecting orbitofrontal and temporal cortices), will also mediate referential communication.

In sum, we hypothesize (1) that patients with bvFTD will have difficulty coordinating perspectives with a conversational partner during referential communication; (2) that impairment will be related to some, but not all, domains of executive function; and (3) that impairment will be related to disease in a prefrontal, social-executive network.

## Materials and Methods

### Participants

Participants included 20 patients with bvFTD (16 male) and 20 healthy control subjects (14 male) who were demographically matched for age (*t*_(38)_ = 0.23, *p* = 0.54), education (*t*_(38)_ = 0.74, *p* = 0.47), and gender (χ^2^(1) = 0.13, *p* = 0.72). See Table 1 for a summary of demographic and clinical characteristics. Patients with bvFTD received diagnoses by board-certified neurologists (M.G., D.J.I.) using a consensus procedure and published criteria (Rascovsky et al., 2011). Patients were classified as nonaphasic by clinician judgment (following clinical examination and elicitation of speech samples), and any patients with symptomatic evidence of semantic variant primary progressive aphasia (which can sometimes co-occur with bvFTD) were excluded from our sample. Alternative causes of cognitive difficulty (e.g., Alzheimer’s disease, hydrocephalus, stroke, or head trauma) were excluded by clinical examination, neuroimaging, and blood tests. As shown in Table 1, dementia severity in patients was assessed using the clinical dementia rating, global score (CDR-Global Score; Morris, 1993), as modified by the inclusion of two FTD-related scales (Knopman et al., 2011). Derived from a semi-structured patient interview, the global CDR assesses functional impairment across six domains using the following 3-point scale: memory, orientation, judgment and problem solving, community affairs, home and hobbies, and personal care. This is supplemented by domains querying language and social functioning. All patients scored in the mild-to-moderate range (range, 0.5–2; mean, 1.075). Informed consent was obtained from all participants according to a protocol approved by the University of Pennsylvania’s Institutional Review Board.

**Table 1.** Mean (±SD) demographic information for behavioral variant frontotemporal degeneration and control groups

	All subjects	
	Patients (*N* = 20)	Healthy Seniors (*N* = 20)
Age (years)	64.65 (9.10)	64.00 (11.05)
Education (years)	16.30 (2.77)	15.75 (2.12)
Disease duration	4.50 (3.24)	N/A
Clinical disease rating, global score	1.08 (0.54)	N/A
	Patient subgroups	
	Poor (*n* = 10)	Good (*n* = 10)
Age (years)	63.00 (4.52)	66.30 (12.18)
Education (years)	16.00 (3.13)	16.60 (2.50)
Disease duration (years)	3.80 (1.62)	5.2 (4.28)
Clinical disease rating, global score	1.3 (0.48)	0.85 (0.53)

N/A, Not applicable.

It is widely accepted that bvFTD is a heterogeneous disorder, with patients showing different behavioral profiles and corresponding patterns of neural atrophy. Further inspection of individual patient profiles (e.g., examination of group boxplots and *z*-scores) suggested the presence of two subgroups within our patient sample. Accordingly, we rank ordered our patients on the basis of overall performance (precise responses in the sighted and color-blind conditions, combined, see below) and divided the sample into two equal subgroups using a median split procedure. The resulting patient subgroups were matched for age (*U* = 43.00, *p* = 0.63), education (*U* = 40.50, *p* = 0.481), CDR-Global Score (*U* = 26.00, *p* = 0.075), and disease duration (*U* = 44.00, *p* = 0.684; Table 1).

### Neuropsychological battery

Patients were administered a targeted neuropsychological battery including both language and executive measures. Language measures included the Boston naming test (BNT; Kaplan et al., 1983), a confrontational naming test using a series of line drawings that are graded in difficulty, and a semantic word-picture matching task (Rogalsky et al., 2011), in which subjects are asked to identify which of two pictures represents a given word. These measures were normalized to 100 and subsequently averaged to yield the language composite. Executive measures were chosen to probe the three postulated subdomains of executive function, as described by Miyake et al. (2000). Mental set shifting was assessed using the visual-verbal test (VVT; Feldman and Drasgow, 1960), which requires subjects to identify two unique but partially overlapping groupings from a given set. Information monitoring and updating (i.e., working memory) was assessed using the backward digit span (Weschler, 1997), which requires subjects to repeat an orally presented sequence of numbers in reverse order. Finally, the inhibition of prepotent responses was assessed using the disinhibition subscore of the neuropsychiatric inventory (NPI-Disinhibition), which is a caregiver-based instrument assessing the frequency and severity of neuropsychiatric symptoms in a patient (Cummings, 1997). Finally, perspective-taking ability was assessed using the perspective-taking subscore of the interpersonal reactivity index, an informant-based measure with 28 items assessing four domains of empathy (empathic concern, personal distress, fantasy, and perspective taking; Davis, 1983).

### Experimental design and statistical analyses

**Figure 1.**
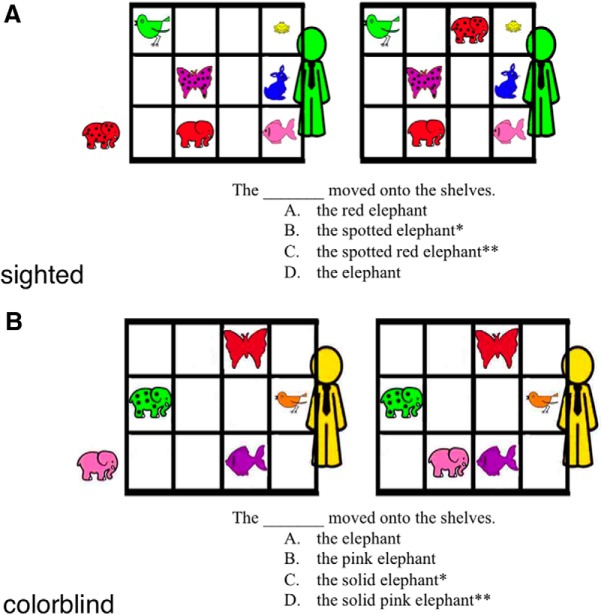
Experimental design. Participants were presented two-scene stories in which a target animal moves from one location to another. ***A***, ***B***, Above are two stimuli from two conditions (***A***, sighted; ***B***, color blind). The stimuli also vary in the number of competitors (i.e., animals of the same species as the target) visible in the scene (***A*** and ***B*** both have 1 competitor). Participants were presented with a multiple-choice item below each stimulus, consisting of a probe and four fill-in-the-blank answer choices. Participants were asked to select the correct response that would identify the target (for the avatar visible behind the shelves) while using the minimal numbers of descriptors. The precise response for each trial is denoted with a single asterisk (*), and the superfluous response with a double asterisk (**).

Participants were presented with brief, two-scene stories illustrating the movement of a target cartoon animal. On each trial, the target animal was moved from the floor to a shelf (i.e., a three by four array) of objects that shared some combination of color, size, and pattern features with the target (Fig. 1). The participants’ task was to successfully communicate which animal in the final array had been moved. An avatar visible behind the shelves represented a human interlocutor. Participants were given a multiple-choice selection of adjectives below each stimulus to describe the target animal that had been moved. The multiple-choice selection consisted of a probe and four fill-in-the-blank answer choices. Participants were instructed to select the best response that would correctly distinguish the target animal, without using any unnecessary descriptors. Answer choices varied in the number and type of adjectives, but all answers referenced the correct species of animal. Answer choices were classified according to the following response types: precise, superfluous, insufficient, irrelevant, and violations. Precise responses constitute the optimal response, using only those adjectives that are necessary and no additional adjectives. Superfluous responses, while accurate, were defined as responses using an excess number of adjectives and therefore requiring gratuitous effort by both speaker and listener (e.g., for the target “red pig,” selecting “big red pig”). Insufficient responses are responses using too few adjectives (e.g., for the target red pig, selecting “pig”). In practice, insufficient responses lead to ineffective communication, as the avatar would be unable to identify the target animal. Irrelevant responses used the correct number of adjectives, but like insufficient responses, lacked critical adjectives necessary to distinguish the target object from its competitors (e.g., for the target red pig, selecting “big pig” although all the pigs in the array are the same size). Finally, violations used a factually incorrect adjective (e.g., for the target red pig, selecting “yellow pig”).

Trials varied in two dimensions. First, to manipulate perspective-taking demand, trials varied in the amount and type of information accessible to the avatar. In “sighted” trials, the avatar had full access to visual information. The sighted condition is thus analogous to the common ground condition in previous research. In color-blind trials, on the other hand, the avatar was described as color blind and seeing only in black-and-white/grayscale. In each of the color-blind trials, the target animal was unique in at least two dimensions, one of which was always color. Therefore, to correctly identify the target animal, the subject would have to reject the color-based answer in favor of the answer that referred to shape and/or size. This condition is thus analogous to a “privileged ground” condition, where there is unequal access to information between the two partners that must be taken into account when selecting the appropriate attributes. Our color-blind condition, however, better mirrors the type of perspective taking typically engaged in daily conversation, compared with the traditional privileged ground conditions that involve a physical obstruction and significant visuospatial processing.

Second, to manipulate working memory demands, the stimuli also differed according to the number of competitors (i.e., animals of the same species; e.g., “pigs” or “elephants”) present in the array (0, 1, or 3). The number of competitors visible in the array thus determined the number of adjectives necessary for the participant to adequately distinguish the target animal when describing its movement to the avatar. Please see Figure 1 for an illustration of stimulus materials.

There were eight stories for each level of competitor for each condition, with a total of 48 stimuli equally distributed across conditions. Stimuli were presented in a pseudo-randomized order to ensure that a single condition (e.g., color-blind, three competitors) was not repeated across consecutive trials. Repetition of trial type could encourage perseveration or the formation of alternative strategies and heuristics, rather than on-line perspective taking. To ensure the comprehension of task instructions, subjects were trained before testing and given feedback on practice items. All participants appeared to understand the task. In total, task administration took ∼1 h.

Subject responses were digitally recorded and classified according to the following response types: precise, superfluous, insufficient, irrelevant, or violation. Individual subject scores were then generated by dividing the number of responses given for each type (e.g., precise, superfluous) by the total number of trials in the given condition (or the experiment as a whole, as appropriate). Precise and superfluous responses were also summed to create an overall accuracy score, since both types of responses would allow the avatar to correctly identify the target animal.

We used nonparametric statistics to analyze behavioral performance, as all data were not normally distributed according to Shapiro–Wilk tests. Between-group comparisons used Mann–Whitney tests, and within-group comparisons used Wilcoxon tests. Correlation analyses were calculated using the Spearman method. All results were corrected for multiple comparisons using the Bonferroni procedure.

### Structural imaging procedure and analysis

High-resolution volumetric T1-weighted MRI was available for 18 patients with bvFTD (note: these 18 patients did not differ from the original 20-patient cohort for all demographic variables and experimental outcomes; all *p* values >0.5). MRI images were not available for two individuals with bvFTD (one “good” performer and one “poor” performer) for health and safety reasons, including claustrophobia and metallic implants (e.g., pacemakers, shrapnel) in the body. MRI volumes were acquired using an MPRAGE sequence from a SIEMENS 3.0 T Trio scanner with an eight-channel head coil and the following acquisition parameters: repetition time (TR), 1620 ms; echo time (TE), 3.87 ms; slice thickness, 1.0 mm; flip angle, 15°; matrix, 192 × 256; and in-plane resolution, 0.9766 × 0.9766 mm. Whole-brain MRI volumes were preprocessed using Advanced Normalization Tools (https://github.com/ANTsX/ANTs) using the state-of-the-art antsCorticalThickness pipeline described previously (Avants et al., 2008; Klein et al., 2010; Tustison et al., 2014). Briefly, processing begins by deforming each individual dataset into a standard local template space. A diffeomorphic deformation was used for registration that is symmetric to minimize bias toward the reference space for computing the mappings, and topology preserving to capture the large deformation necessary to aggregate images into a common space. Template-based priors are used to guide GM segmentation and to compute GM probability, which reflects a quantitative measure of GM density. Resulting images are warped into MNI space, smoothed using a 2 σ smoothing kernel and downsampled to 2 mm resolution, which best reflects the average cortical thickness across the brain and is often required to achieve statistical significance.

Permutation-based imaging analyses were performed using the randomise tool in FSL (http://fsl.fmrib.ox.ac.uk/fsl/fslwiki). Briefly, permutation-based *t* tests evaluate a true assignment of GM density across groups (signal) relative to many (10,000) random assignments of GM density across groups (noise) and thus is a statistically robust procedure that is much less susceptible to multiple-comparison problems compared with traditional parametric-based *t* tests (Winkler et al., 2014). GM density was compared in patients relative to an independent cohort of 36 healthy age- and education-matched control subjects from the surrounding community (age: mean = 62.36, SD = 7.35, *p* = 0.4117; education: mean = 15.79, SD = 2.17, *p* = 0.36). Analyses were run with 10,000 permutations and restricted to voxels containing GM using an explicit mask generated from the average GM probability map of all groups. We report clusters that survived a threshold of *p* < 0.001, correcting for multiple comparisons using the familywise error (FWE) rate and threshold-free cluster enhancement (Smith and Nichols, 2009) . Results were projected onto the Conte69 surface-based atlas using Connectome Workbench (http://www.humanconnectome.org/software/connectome-workbench.html).

To relate behavioral performance to regions of significant GM disease, we conducted regression analyses with the randomise tool of FSL. Permutations were run exhaustively up to a maximum of 10,000 for each analysis. To constrain our interpretation to areas of known GM disease, we restricted our regression analyses to an explicit mask containing voxels of GM atrophy in the patients as defined in the above group comparison. Results outside these regions of atrophy would be difficult to interpret since they could be attributed to a variety of factors not related to disease (e.g., healthy aging, genetic differences). We report clusters surviving a height threshold of *p* < 0.005 and a minimum of 10 adjacent voxels, a joint threshold suggested for the optimal balance of type I and type II error rates (Lieberman and Cunningham, 2009). Covariates were run separately for the sighted condition, color-blind condition, and visual-verbal test. Conjunction analyses were conducted in FSL to identify significant regions that were common to different tasks (i.e., sighted and VVT, color blind and VVT). All regression results were projected onto slices using MRIcron software (Rorden and Brett, 2000).

### Diffusion tensor imaging procedure and analysis

Diffusion tensor imaging was available for the same 18 patients with bvFTD with T1 imaging. A 30-directional diffusion-weighted imaging (DWI) sequence was collected using single-shot, spin-echo, diffusion-weighted echoplanar imaging (FOV, 240 mm; matrix size, 128 × 128; number of slices, 70; voxel size, 1 mm isotropic; TR, 8100 ms; TE, 83 ms; fat saturation). Thirty volumes with diffusion weight (b = 1000 s/mm^2^) were collected along 30 noncollinear directions, and either one or five volumes without diffusion weight (b = 0 s/mm^2^) were collected per subject. The PipeDream processing pipeline used ANTs (Tustison et al., 2014) and Camino (Cook et al., 2006) to preprocess DWI. Motion and distortion artifacts were removed using affine coregistration of each diffusion-weighted image to the average of the unweighted (b = 0) images. Diffusion tensors were calculated using a weighted linear least-squares algorithm (Salvador et al., 2005) implemented in Camino. Fractional anisotropy (FA) was computed in each voxel from the DT image, and distortion between the subject’s T1 and DT image was corrected by registering the FA to the T1 image. DTs were then relocated to the local template for statistical analysis by applying the FA-to-T1 and T1-to-local template warps, and tensors were reoriented using the preservation of the principal direction algorithm (Alexander et al., 2002). Each participant’s FA image was recomputed from the DT image in local template space and smoothed using a 2 σ smoothing kernel.

Like the pipeline for GM analysis, we used the randomise tool in FSL to compare FA in patients relative to the same cohort of healthy age-matched control subjects. The two-sample *t* test of patients versus control subjects was run with 10,000 permutations and restricted to voxels containing WM based on an explicit mask of high probability WM (minimum FA considered WM, 0.25). Results were again thresholded at *p* < 0.001 and corrected for multiple comparisons using FWE and threshold-free cluster enhancement. Regression analyses then related patient impairment to reduced FA, using covariates for sighted, color-blind, and VVT performances, respectively. These regressions were restricted to the results of the previous analysis—that is, only voxels showing a significant effect of group. Consistent with the GM analyses, we report only clusters surviving a height threshold of *p* < 0.005 and a minimum of 10 contiguous voxels.

## Results

### Behavioral results in patients

**Figure 2.**
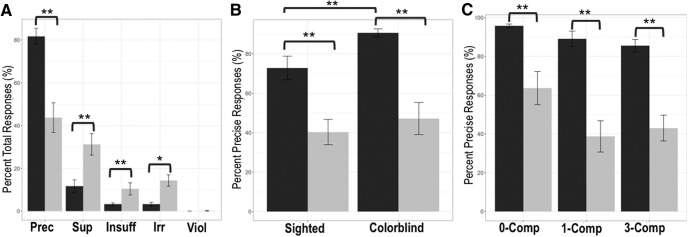
Behavioral performance in patients with behavioral variant frontotemporal degeneration. ***A***, Distribution of mean (±SEM) responses, collapsed across all conditions. Prec, Precise; Sup, superfluous; Insuff, insufficient; Irr, irrelevant; Viol, violation. Patients select significantly fewer precise responses than healthy control subjects and significantly more superfluous, insufficient, and irrelevant responses. There is no group difference in violations. ***B***, Mean (±SEM) precise responses in sighted and color-blind conditions. Patients select significantly fewer precise responses than healthy seniors in both conditions. ***C***, Mean (±SEM) precise responses in 0-competitor, 1-competitor, and 3-competitor conditions. Patients select significantly fewer precise responses than healthy control subjects in all three conditions. For all figures, healthy control subjects are shown in dark gray and bvFTD patients are shown in light gray. *Significant at *p* < 0.05; **significant at *p* < 0.01.

To examine overall performance in patients, we first examined the distribution of response types, collapsed across both sighted and color-blind conditions (Fig. 2*A*). For the purposes of this analysis, we excluded 0-competitor trials as filler trials and only looked at 1-competitor and 3-competitor trials. These 0-competitor trials, which serve as a baseline measure, are excluded because they do not include all types of response types (note: “insufficient” responses do not exist for 0-competitor trials. Because 0-competitor trials only require that the animal species be named correctly, no modifying adjective is necessary, and insufficient responses with too few adjectives are an impossibility). Here, we found that patients selected significantly fewer precise responses than matched control subjects (*U* = 67.00, *p* < 0.001). Instead, patients opted for significantly more superfluous responses (*U* = 92.00, *p* = 0.003). It is important to note that while superfluous responses are not the optimal strategic choice, they still do provide the requisite information needed for a communicative partner to correctly identify the target animal. Patients with bvFTD also selected significantly more insufficient and irrelevant responses than healthy control subjects, both of which lack critical, discriminating adjectives (insufficient: *U* = 96.00, *p* = 0.004; irrelevant: *U* = 93.50, *p* = 0.03). Finally, as expected, patients and healthy control subjects did not differ in regard to violations (*U* = 190.00, *p* = 0.799). This null result for violations suggests that any deficit observed in patients with bvFTD is not due to a baseline language or perceptual impairment. Additionally, to confirm that age, disease severity, and language ability did not account for the decrement in patient performance, we calculated Spearman correlations for performance within each condition (sighted, color blind) with age, disease duration, and language composite score (combined score generated from BNT and word–picture matching). No significant correlations were observed (Table 2). Considered as independent metrics, our language measures also confirm that our patients with bvFTD are nonaphasic: patients scored within normal limits on both the BNT (mean, 25.35 of 30) and word–picture matching (mean, 19.89 of 20). Together, these data suggest that patient impairment is due to the social and/or executive characteristics of the disease, rather than overall cognitive status or language ability. Finally, we also correlated performance in each condition with the perspective-taking subscore of the interpersonal reactivity index, finding a positive association for the color-blind condition but not the sighted condition. These data (Table 2), showing the ability to adapt to the characteristics of the avatar is dependent on general perspective-taking ability, and support the ecological and construct validity of our task.

**Table 2.** Correlations of age, disease duration, language composite, and perspective-taking score with performance in the sighted and color-blind conditions

	Mean (SD)	Sighted	Color blind
Age (years)	64.65 (9.10)	ρ = 0.21, *p* = 0.37	ρ = 0.21, *p* = 0.37
Disease duration (years)	4.50 (3.24)	ρ = 30, *p* = 0.19	ρ = 0.14, *p* = 0.56
Language composite	92.04 (6.72)	ρ = 0.12, *p* = 0.63	ρ = 0.32, *p* = 0.20
Perspective taking (IRI)	17.75 (18.75)	ρ = −0.24, *p* = 0.36	ρ = −0.55, *p* = 0.02^*^

* Significant at *p* < 0.05. IRI: Interpersonal Reactivity Index.

For subsequent analyses, we focused on the precise response type, which represents the optimal selection choice. Here, we observed that patients with bvFTD offered significantly fewer precise responses than control subjects in both the sighted and color-blind conditions (sighted: *U* = 88.50, *p* = 0.002; color blind: *U* = 72.00, *p* < 0.001; Fig. 2*B*). In healthy control subjects, a within-group comparison showed that there is a significant modulation of performance by condition, such that the rate of precise responses is significantly greater in the color-blind condition (*z* = −2.98, *p* = 0.003). This suggests that healthy control subjects engage in active perspective-taking behavior and use the additional information provided to them in the color-blind condition to improve their selection process. In contrast, patients show no difference in performance across the sighted and color-blind conditions (*z* = −1.51, *p* = 0.13).

Next, we considered the potential modulatory effect of competitor number on performance (Fig. 2*C*). We interpret the increase in competitor number as a manipulation of working memory demand, since there are more objects to be maintained and manipulated during the visual search. Here, we found that patients select significantly fewer precise responses than healthy control subjects in all three conditions (0-competitor: *U* = 71.00, *p* < 0.001; 1-competitor: *U* = 77.00, *p* = 0.001; 3-competitor: *U* = 73.00, *p* < 0.001). While this analysis thus suggests that patients are impaired even in the baseline 0-competitor condition, we point out that patients with bvFTD perform at ceiling in regard to accuracy (a combination of precise and superfluous responses, both of which allow the conversational partner to correctly identify the target animal; mean accuracy, 99.06; SD, 3.06). Additionally, in healthy control subjects, although performance is consistently above chance, a Friedman test indicated that performance worsens with increasing competitor number (χ^2^(2) = 21.30, *p* < 0.001). Patients showed no such modulation (χ^2^(2) = 0.11, *p* = 0.95), suggesting that working memory demands are not responsible for the decrement in patient performance.

### Neuropsychological correlations

Next, to examine the neuropsychological mechanism underlying the observed patient deficits, we administered a targeted neuropsychological battery probing the following three domains of executive function, as described by Miyake et al. (2000): mental set shifting, information updating (i.e., working memory), and inhibition. These were assessed using the visual-verbal test, backward digit span, and NPI-Disinhibition, respectively. We used Spearman correlations to relate each of these measures to performance in the sighted and color-blind conditions, separately. While patient performance was not significantly different between these conditions, it remains possible that different mechanisms underlie performance in each. In line with the earlier behavioral findings (i.e., no effect of competitor number), results indicated that working memory capacity was not related to performance in either the sighted or color-blind condition. The same null result was found for NPI-Disinhibition. Mental set shifting, however, as assessed by the visual-verbal test, showed a robust effect and was positively correlated with performance in both conditions, suggesting a specific role for mental set shifting in referential communication (Table 3, all neuropsychological data).

**Table 3.** Correlations of the three executive function subdomains with performance in the sighted and color-blind conditions

	Mean (SD)	Sighted	Color blind
Backward digit span (/8)	4.15 (1.27)	ρ = 0.44, *p* = 0.07	ρ = 0.35, *p* = 0.16
Visual-verbal test (/10)	7.79 (2.51)	ρ = 0.85, *p* < 0.001^**^	ρ = 0.59, *p* = 0.006^**^
NPI-Disinhibition (/3)	1.875 (2.16)	ρ =0.16, *p* = 0.442	ρ = 0.049, *p* = 0.81

** Significant at *p* < 0.01.

### Subgroup analyses in patients

**Figure 3.**
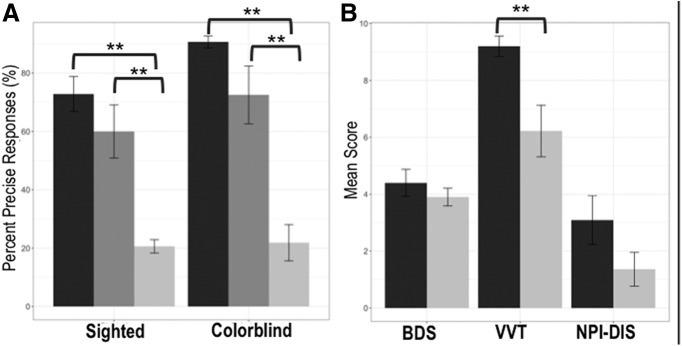
Behavioral and neuropsychological performance across subgroups. ***A***, Mean (±SEM) precise responses in common ground and color-blind conditions for healthy control subjects (dark gray), good performers (medium gray), and poor performers (light gray). Poor performers select significantly fewer precise responses than good performers and healthy control subjects in both conditions. ***B***, Mean backward digit span (BDS), VVT, and NPI-Disinhibition Subscore (NPI-DIS) scores (±SEM) in good performers (dark gray) and poor performers (light gray). While there are no significant differences in BDS or NPI-DIS, good performers have significantly better VVT scores than poor performers. **significant at *p* < 0.01.

Next, we divided the patient cohort into two subgroups (good performers and poor performers) on the basis of a median split, using the overall precise responses performance metric. Within the sighted condition, a Kruskal–Wallis test indicated that there were significant differences among the three groups (H(2) = 17.71, *p* < 0.001). *Post hoc* comparisons revealed no differences between the healthy control subjects and the good performers (sighted: *U* = 128.5, *p* = 0.215; color blind: *U* = 123.5, *p* = 0.293). In contrast, the poor performers were significantly worse than the control subjects (sighted: *U* = 182, *p* < 0.001; color blind: *U* = 198.5, *p* < 0.001) and also significantly worse than the good performers, as expected (sighted: *U* = 1.50, *p* < 0.001; color blind: *U* = 8.50, *p* = 0.001). Figure 3*A* shows a visual depiction of results. To confirm that the differences in performance across subgroups were not a result of differential language ability, we also computed linear models. For both the sighted and color-blind models (run separately), group was a significant predictor (sighted: β = −38.19, *p* = 0.002; color blind: β = −50.13, *p* = 0.003) while language composite was not (sighted: β = −70.19, *p* = 0.40; color blind: β = −64.14, *p* = 0.57).

To understand why some patients were able to maintain performance at normal levels while others were not, we next compared the good and poor performers on the basis of their executive functioning. These data are consistent with the earlier correlation analyses: there was no significant difference between subgroups in regard to backwards digit span (*U* = 60.00, *p* = 0.49) or NPI-Disinhibition (*U* = 37.00, *p* = 0.58), but there was a highly significant difference in regards to visual-verbal test (*U* = 80.00, *p* < 0.003; Fig. 3*B*).

### Structural MRI results in all patients

**Figure 4.**
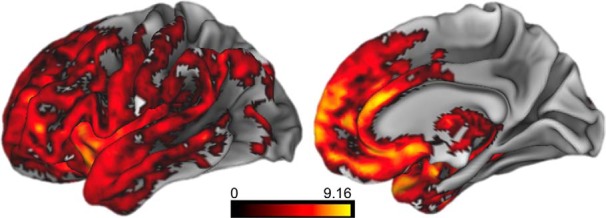
Surface renderings depicting regions of significantly reduced gray matter density in bvFTD patients relative to age-matched healthy control subjects. Heat map intensity refers to *t* statistic value.

**Figure 5.**
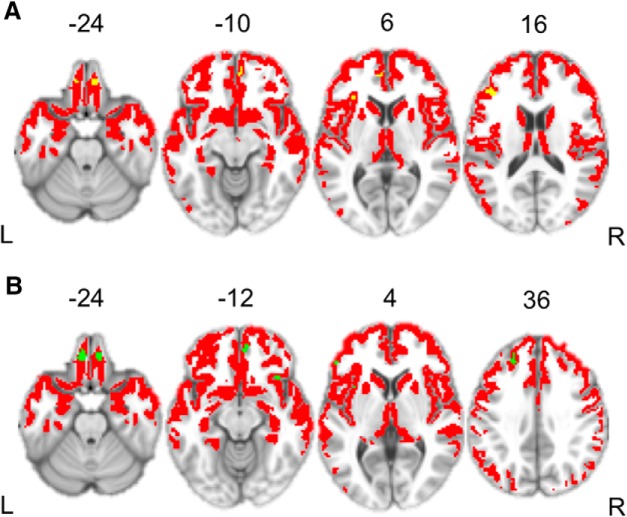
Structural imaging results. ***A***, Yellow regions represent regions of reduced gray matter density related to performance in sighted trials. ***B***, Green regions represent regions of reduced gray matter density related to performance in color-blind trials. ***A***, ***B***, Red regions represent areas of significantly reduced gray matter density in patients with behavioral variant frontotemporal degeneration relative to healthy control subjects.

Next, we addressed the question of whether atrophy in patients with bvFTD is related to impaired social coordination during referential communication. First, we contrasted GM density in patients with bvFTD relative to an independent cohort of healthy control subjects. As expected, this analysis revealed significantly reduced GM density throughout the frontal lobes and anterior temporal lobes in patients with bvFTD, which is consistent with disease diagnosis (Fig. 4, Table 4). To relate discourse deficits to GM density, we performed a regression analysis in the patient group, using the percentage of precise responses in the sighted and color-blind conditions as covariates in two separate analyses. The results were largely consistent across conditions, with similar effects found in the mPFC, orbitofrontal cortex (OFC), and dorsolateral prefrontal cortex (DLPFC), as well as insula. Within DLPFC, Brodmann area (BA) 46 was related to performance in the sighted condition, while BA 9, which is anterior to BA 46, was related to color-blind performance. Finally, the color-blind condition also shows a unique relationship with inferior frontal gyrus pars triangularis (Fig. 5, Table 5).

**Table 4.** Peaks and subpeaks of reduced gray matter density in patients with behavioral variant frontotemporal degeneration

	L/R	MNI coordinates			*t* Statistic	Voxels
Neuroanatomic region (BA)		*x*	*y*	*z*		
Clusters of reduced gray matter density in patients with bvFTD relative to control subjects						
Temporal pole (38)	L	−30	16	−32	9.16	37,306
Inferior frontal gyrus (47)	L	−24	12	−22	8.50	Sub
Insular cortex (13)	L	−32	24	6	8.43	Sub
Orbitofrontal cortex (47)	L	−30	34	−16	8.27	Sub
Anterior cingulate cortex (32)	R	0	36	14	8.15	Sub
Medial prefrontal cortex (11)	R	2	44	−16	7.75	Sub

MNI, Montreal Neurologic Institute; Sub, subpeak; R, right; L, left.

**Table 5.** Regions of reduced gray matter density in patients with behavioral variant frontotemporal degeneration related to performance in sighted, color-blind, and visual-verbal test

	L/R	MNI coordinates			*t* Statistic	Voxels
Neuroanatomic region (BA)		*x*	*y*	*z*		
Clusters of reduced gray matter density related to sighted performance						
Dorsolateral prefrontal cortex (46)	L	−44	36	18	4.19	41
Medial prefrontal cortex (11)	R	6	52	−14	3.59	21
Orbitofrontal cortex (11)	R	10	44	−24	4.23	19
Orbitofrontal cortex (11)	L	−10	44	−26	3.93	14
Insula	L	−32	30	6	3.63	14
Anterior cingulate cortex (32)	L	−4	50	4	3.40	10
Clusters of reduced gray matter density related to color-blind performance						
Orbitofrontal cortex (11)	L	−8	46	−24	4.51	34
Medial prefrontal cortex (11)	R	6	52	−14	4.80	26
Orbitofrontal cortex (11)	R	10	44	−22	4.24	24
Dorsolateral prefrontal cortex (9)	L	−22	40	38	3.89	17
Insula	L	−36	18	6	3.47	15
Inferior frontal gyrus (45)	L	−52	38	2	5.66	11
Insula	R	36	24	−10	3.49	10
Clusters of reduced gray matter density related to visual-verbal test performance						
Orbitofrontal cortex (11)	R	10	42	−24	5.48	97
Insula	R	36	28	−4	5.63	84
Orbitofrontal cortex (11)	R	22	32	−14	6.42	84
Orbitofrontal cortex (11)	L	−12	40	−24	5.44	80
Insula	R	32	18	6	5.02	53
Caudate nucleus	R	10	10	6	5.17	52
Insula	L	−32	14	4	4.01	41
Insula	R	40	8	2	3.94	39
Inferior frontal gyrus (45)	L	−30	30	8	3.75	39
Inferior frontal gyrus (46)	L	−42	34	16	3.74	33
Caudate nucleus	L	−10	14	2	4.46	22
Medial prefrontal cortex (10)	R	14	44	10	4.86	18
Anterior cingulate cortex (32)	L	−4	50	4	4.58	18
Dorsolateral prefrontal cortex (9)	R	40	32	18	4.04	12
Inferior frontal gyrus (46)	L	−46	46	4	4.04	10

MNI, Montreal Neurologic Institute; R, right; L, left.

**Figure 6.**
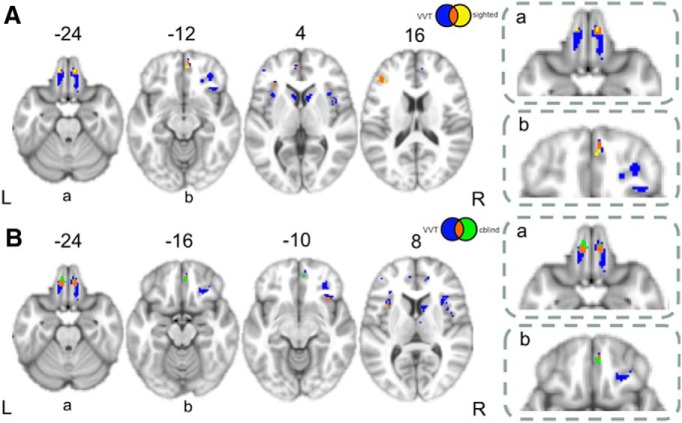
Conjunction Analysis. ***A***, Conjunction of sighted (yellow) and VVT (blue) regressions. Overlap (i.e., regions where both tasks are significantly associated) is shown in orange. ***B***, Conjunction of color-blind (“cblind”, green) and VVT (blue) regressions. Overlap is again shown in orange. See insets for close-up views of conjunction results in orbitofrontal (labeled a) and medial prefrontal cortex (labeled b).

Given the neuropsychological findings and the purported role of mental set shifting in referential communication, we also related performance on the visual-verbal test to GM atrophy (Table 5), and compared this to our previous imaging findings from both the sighted and color-blind conditions. Any overlap (sighted-VVT or color blind-VVT) could be interpreted as evidence of a common neural correlate across tasks. Indeed, for the sighted condition, we found overlapping results throughout prefrontal cortex: OFC, mPFC, and DLPFC. Similarly, overlapping results between the visual-verbal test and color-blind performance were found in portions of OFC, mPFC, and insula. These data again suggest that referential communication involves mental set shifting (Fig. 6, conjunction results, Table 6 conjunction results).

**Table 6.** Conjunction analysis with peak coordinates representing regions of overlap between sighted and VVT performance and color-blind and VVT performance

	L/R	MNI coordinates			Voxels
Neuroanatomic region (BA)		*x*	*y*	*z*	
Conjunction of sighted and VVT performance					
Dorsolateral prefrontal cortex (46)	L	−42	38	12	23
Orbitofrontal cortex (11)	R	10	42	−24	16
Orbitofrontal cortex (11)	L	−6	42	−28	13
Insula	L	−34	28	2	10
Anterior cingulate cortex (32)	L	−2	50	2	7
Medial prefrontal cortex (11)	R	6	52	−14	5
Conjunction of color-blind and VVT performance					
Orbitofrontal cortex (11)	L	−8	40	−28	26
Orbitofrontal cortex (11)	R	10	40	−24	21
Insula	L	−34	14	4	13
Insula	R	38	22	−12	10
Medial prefrontal cortex (11)	R	6	50	−16	7

MNI, Montreal Neurologic Institute; R, right; L, left.

### Diffusion tensor imaging results in all patients

**Figure 7.**
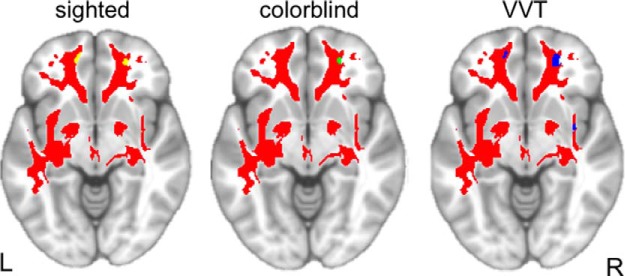
Slices depicting regions of reduced fractional anisotropy in patients with bvFTD compared with healthy control subjects (red), overlaid by regions of reduced FA significantly related to task performance, as labeled. Sighted, yellow; color blind, green; VVT, blue. For all slices, *z* = −7.

To our knowledge, the majority of studies on referential communication and other examples of language-based coordination tasks have focused primarily on the role of GM regions. In the current study, we adopted a multimodal approach and also collected high-resolution diffusion tensor imaging to examine the possible involvement of white matter projections across the brain. As illustrated in Figure 7 and Table 7, we found significantly reduced FA in portions of the corticospinal tract, corpus callosum, inferior longitudinal fasciculus, and uncinate fasciculus in patients with bvFTD relative to healthy age-matched control subjects. In a series of *post hoc* regression analyses, we identified significant associations between FA and performance in the uncinate fasciculus and corpus callosum, for each sighted, color-blind, and VVT performance. FA was also significantly associated with color-blind performance in WM of inferior frontal gyrus and with VVT performance in the inferior fronto-occipital fasciculus.

**Table 7.** Diffusion tensor imaging results: white matter tracts and peak coordinates of significantly reduced fractional anisotropy in bvFTD patients compared with healthy control subjects and regression of task performance with regions of reduced fractional anisotropy for sighted condition, color-blind condition, and VVT

	L/R	MNI coordinates			*t* Statistic	Voxels
White matter projection		*x*	*y*	*z*		
Regions of reduced FA in patients versus control subjects (red)						
Corticospinal tract	L	−18	−5	−11	8.53	92,123
Corpus callosum (frontal)	R	11	22	16	6.80	sub
Corpus callosum (frontal)	L	−6	23	−2	6.77	sub
Uncinate fasciculus	L	−28	5	−11	6.76	sub
Inferior longitudinal fasciculus	L	−33	−14	−15	6.67	sub
Fusiform gyrus WM	L	−23	−22	−19	6.32	350
Regions of reduced FA related to sighted performance in patients (yellow)						
Uncinate fasciculus	L	−12	54	−5	5.75	114
Uncinate fasciculus	R	23	47	−7	5.87	37
Corpus callosum (frontal)	L	−15	49	5	4.17	17
Regions of reduced FA related to color-blind performance in patients (green)						
Inferior frontal gyrus WM	L	−47	16	19	3.93	31
Inferior frontal gyrus WM	L	−45	29	11	3.23	24
Uncinate fasciculus	R	23	47	−7	3.37	23
Corpus callosum (frontal)	L	−12	54	−5	3.28	12
Regions of reduced FA related to VVT performance in patients (blue)						
Uncinate fasciculus	R	23	46	−6	4.74	184
Superior frontal gyrus WM	L	−17	49	7	3.41	170
Uncinate fasciculus	L	−15	51	−6	3.39	108
Superior frontal gyrus WM	R	19	53	1	5.74	77
Inferior fronto-occipital fasciculus	R	35	−1	−7	3.58	66
Superior temporal gyrus WM	L	−47	−19	1	3.54	30
Superior temporal gyrus WM	L	−47	−6	−13	3.11	19
Corpus callosum (frontal)	R	3	28	5	4.97	14

MNI, Montreal Neurologic Institute; Sub, subpeak; R, right; L, left.

## Discussion

This study examined the cognitive and neuroanatomic bases of impaired referential communication in patients with bvFTD. Using a carefully controlled experimental task, subjects were asked to indicate the movement of a target object to an interlocutor, who shared a variable amount of access to visual information. We found that bvFTD patients have deficits coordinating perspectives with a partner, due in part to deficits in mental flexibility and disease in a prefrontal network associated with social and executive functioning. These findings highlight the essential contribution of nonlanguage brain regions to referential communication. We discuss our cognitive and anatomic results in turn below.

### Referential communication is impaired in bvFTD

Overall, the behavioral results across both sighted and color-blind conditions indicated that bvFTD patients select significantly fewer precise responses than healthy control subjects, instead offering more superfluous, as well as insufficient and irrelevant responses. These results mirror the finding of decreased overall accuracy for patients with bvFTD relative to control subjects on task-monitoring adjective use during overt speech production and using similar stimulus materials (Healey et al., 2015). The patients’ frequent selection of superfluous responses—that is, responses that are overspecified and include excess numbers of adjectives—is considered suboptimal here because they violate the Gricean maxim of quantity (i.e., be as brief as possible; Grice, 1975). While some research has found that the inclusion of extra information during communication can facilitate object identification and promote learning (Maes et al., 2004; Arts et al., 2011), other studies have demonstrated that overspecified referring expressions can actually impair comprehension (Arbuckle et al., 2000; Engelhardt et al., 2006, 2011). For example, in an event-related potential study examining how listeners process object descriptions with unnecessary modifiers (e.g., “red square” in a context where only one square was displayed), healthy participants were slower to orient to the target object when unnecessary modifiers were used (Engelhardt et al., 2011). Furthermore, an N400 effect, thought to index semantic integration difficulties (Kutas and Hillyard, 1980; Kutas and Federmeier, 2011), emerged 200-300 ms after modifier onset.

Unlike superfluous responses, insufficient and irrelevant responses are missing critical, discriminating adjectives that would be necessary for the interlocutor to correctly identify the target. In a real-world context, these responses, which patients with bvFTD selected more frequently than control subjects, would constitute a failure to communicate effectively. Importantly, patients did not select more violation responses than healthy control subjects, indicating that lexicosemantic or visuospatial deficits are unlikely to contribute to impaired performance. It is unlikely that language impairment can explain the bvFTD deficit, as they are nonaphasic and generally demonstrate relatively preserved language skills. Indeed, when we tested for a relationship between our composite language score and performance on the coordination task, we found no significant effect.

Interestingly, healthy control subjects, who showed performance well above chance in both conditions, showed significantly better performance for color-blind trials compared with sighted trials. This pattern suggests that healthy control subjects acted strategically and used the condition information to eliminate incorrect options using color terms (and thereby increased the chance of a precise response). Such an explanation aligns well with the previous literature showing that older adults tend to show an increased use of heuristics when problem solving compared with younger adults, who rely more on on-line analytical reasoning (Johnson, 1990; Klaczynski and Robinson, 2000; Kim and Hasher, 2005; Worthy and Maddox, 2012; Rydzewska et al., 2018). For example, using a computer-based sequential choice task, Rydzewska et al. (2018) found that older adults adopt compensatory strategies during complex decision-making tasks and reduce the number of options they consider over time, without sacrificing performance.

Patients with bvFTD, on the other hand, were insensitive to the difference between conditions and did not modulate their use of color adjectives in the same manner as healthy control subjects. While the finding that bvFTD patients are not significantly more impaired on color-blind trials may seem counterintuitive, it is not unexpected. We note here that both the sighted and color-blind conditions require perspective taking. The interesting question is not which condition is more difficult, but whether or not patients can adapt their responses to the specific demands of a given trial, using color terms, as appropriate, in sighted trials but never in color-blind trials. Indeed, our data show that patients fail to consider the characteristics of their conversational partner when planning their response—a phenomenon known as recipient design (Blokpoel et al., 2012). The absence of a condition effect in bvFTD again suggests that this group consistently responds in a superfluous way.

### Referential communication is related to mental flexibility

Although previous studies have consistently reported executive deficits in patients with bvFTD (Kramer et al., 2003; Rosen et al., 2004; Libon et al., 2007; Possin et al., 2013; Baez et al., 2019), there has been conflicting evidence regarding the relationship between social cognition and executive function (Lough et al., 2006; Eslinger et al., 2007; Le Bouc et al., 2012; Bertoux et al., 2016). It is possible that these discrepant findings are a result of examining different subdomains of executive function. For example, Eslinger et al. (2007) used the visual-verbal test to probe executive function, while Le Bouc et al. (2012) used the Stroop test, Trailmaking Test, and verbal fluency to assess executive function. In the current study, by specifically probing each of the three postulated domains of executive function described by Miyake et al. (2000), we may be able to resolve this debate in the context of communication. We also explicitly manipulated working memory demands by systematically varying the number of competitors (i.e., objects sharing a perceptual feature with the target object) present in the array. Results showed that while patients were impaired relative to control subjects in the 0-competitor, 1-competitor, and 3-competitor conditions, there was no modulatory effect of competitor number, suggesting a minimal contribution of working memory. It is important to point out here that although patients selected significantly fewer precise responses than control subjects in the baseline 0-competitor condition, they were still highly accurate (accurate responses are either precise or superfluous, both of which allow the interlocutor to correctly identify the target). The persistent use of superfluous descriptors across all conditions is nevertheless suboptimal according to the maxim of quantity of Grice (1975) and is consistent with previous reports in bvFTD documenting speech that is tangential and/or lacks essential meaning (Ash et al., 2006; Barsuglia et al., 2014; Mendez et al., 2017).

If the resource demands associated with increasing competitor numbers does not appear to relate to performance, then what does? We collected a targeted executive battery, including measures of working memory, inhibition, and set shifting, to examine whether any executive resources are related to referential communication. As suggested by the null results of competitor conditions, our neuropsychological data confirm that working memory, as measured by the backwards digit span, is not associated with performance in the sighted or color-blind conditions. These results add to the growing body of evidence that perspective taking does not depend on working memory per se, either in healthy adults (Lin et al., 2010; Cavallini et al., 2013; Healey and Grossman, 2016; Cane et al., 2017) or in patients with bvFTD (Freedman et al., 2013; Bertoux et al., 2016). With some evidence for a positive relationship between working memory and perspective taking also reported for both groups (Fizke et al., 2014; Torralva et al., 2015), more research on this topic is still needed.

Like working memory, we also found no evidence for a relationship between inhibitory control and social coordination. This is somewhat surprising, given that social perspective taking is hypothesized to involve the following two major components: (1) inferring the perspective of the other; and (2) inhibiting one’s own perspective (Leslie et al., 2004, 2005; Samson et al., 2007; Le Bouc et al., 2012). It is possible that the type of inhibitory control measured here by the NPI, which represents limited control of social behavior and comportment (e.g., inhibiting outbursts, inappropriate comments), is functionally distinct from the type of cognitive inhibitory control needed for the prescribed perspective-taking task. Indeed, other studies have claimed that individual differences in inhibition can predict perspective-taking abilities both across the life span (Carlson and Moses, 2001; German and Hehman, 2006; Brown-Schmidt, 2009; Nilsen and Graham, 2009; Long et al., 2018) and in disease states (Le Bouc et al., 2012; Schroeter et al., 2014). We do note, however, that previous work on the relationship between different types of inhibition has suggested that resisting distractor interference (likely what our referential communication task assesses) and inhibition of action (likely what the NPI assesses) are strongly correlated and cluster as a single factor in a latent-variable analysis (Friedman and Miyake, 2004). Regardless, additional work is needed to investigate these discrepant findings, likely using a more representative inhibitory control task, such as the Go/No-Go or Hayling sentence test.

Importantly, we did observe a robust relationship between mental set shifting (i.e., performance on the visual-verbal test) and referential communication ability (both conditions). In confirmation of these results, we also found that our bvFTD subgroups, good and poor performers, were significantly different from one another in mental-set shifting, but not other EFs. Positive results may have been observed here because the visual-verbal test (Feldman and Drasgow, 1960) is particularly appropriate for use in clinical populations: it is brief, nonsocial, nonverbal, and has minimal motor demands (Eslinger et al., 2007; Evans et al., 2015). Other authors have found similar results concerning the role of mental set shifting in social behavior. Eslinger et al. (2007), for example, reported that performance on the visual-verbal test was predictive of social dilemma judgments in patients with bvFTD. Previous studies have also found similar results using alternative tasks, including the Wisconsin card sorting test (Torralva et al., 2009, 2015; Flanagan et al., 2018).

### A multimodal, prefrontal network for referential communication

To examine the neuroanatomic basis for referential communication deficits in bvFTD, we conducted a series of structural imaging analyses. Unlike most previous studies, we examine patterns of both GM atrophy and WM damage to build a large-scale, multimodal network associated with successful social communication. Using a whole-brain approach, we found that patients with bvFTD show widespread reductions in GM density in the frontal and anterior temporal lobes compared with healthy control subjects, with peaks in the left temporal pole, OFC, insula, and anterior cingulate cortex. This pattern of atrophy is consistent with the diagnostic criteria for “probable” bvFTD (Rascovsky et al., 2011).

Subsequent analyses examined the potential role that these frontal and temporal regions may play in referential communication more specifically. Parallel regression analyses (using performance either in the sighted or color-blind conditions) showed largely consistent results across conditions, with positive associations found between performance and GM density in OFC, mPFC, DLPFC, and insula. These results are well aligned with previous observations relating narrative expression (i.e., overt speech) to frontal brain regions (Healey et al., 2015).

Our principal finding suggests primary roles for OFC and mPFC in cortical networks supporting referential communication. Take first OFC, which has been previously implicated in studies of set shifting and cognitive flexibility (Badre and Wagner, 2006; Dajani and Uddin, 2015), as well as a range of social behaviors, including emotion and reward processing (Viskontas et al., 2007). Theories of OFC function suggest that this area contributes broadly to networks for everyday decision-making, including the ability to adapt to new environmental contingencies and reverse previous stimulus–reinforcement associations (Murray et al., 2007; Wallis, 2007). In the current paradigm, then, OFC damage may relate to the patients’ inability to adjust their strategy and use of color-based responses according to the given condition. Offering converging evidence for this interpretation is the finding of a common neural substrate for referential communication and VVT, both associated in part with OFC.

Proximal but dorsal to the observed OFC cluster is mPFC, a region included in networks for self-referential processing, perspective taking, and theory of mind (ToM; Gallagher and Frith, 2003; D’Argembeau et al., 2007; Van Overwalle, 2009). We note here that the observed cluster is located in the ventral portion of mPFC, a location that is sometimes considered overlapping or interchangeable with OFC in the clinical literature (Zald and Andreotti, 2010). Furthermore, some previous research suggests that the mPFC functions may vary in part along a dorsal–ventral axis, such that ventral mPFC is involved in affective ToM and dorsal mPFC in cognitive ToM (Abu-Akel and Shamay-Tsoory, 2011). Given this perspective, additional work is still needed to clarify the organization of mPFC, as our cluster is predominantly ventral but our task predominantly cognitive. Alternatively, our data may be consistent with theories of mPFC suggesting that ventral portions are engaged during the generation of explicit inferences about others (as required here), whereas dorsal portions are engaged during spontaneous or implicit inferences (Van Overwalle, 2009). Regardless, our findings are consistent with previous work demonstrating a role for ventromedial prefrontal cortex in social communication (Gordon et al., 2014; Healey et al., 2015; Spotorno et al., 2015; Stolk et al., 2015).

The observed results in DLPFC are also somewhat nuanced. Broadly speaking, the DLPFC is thought to subserve functions such as working memory, relational complexity, and selection among competing responses (Petrides, 2005; Badre and D’Esposito, 2007; Badre, 2008; Suzuki and Gottlieb, 2013), all of which are relevant to the task here. Although DLPFC was implicated in both the sighted and color-blind conditions, the relevant portions of DLPFC were somewhat unique, as follows: BA 9 for sighted and BA 46 for color blind. These regions are thought to process different types of information. For example, Badre (2008) and Badre and D’Esposito (2007) hypothesized a hierarchical rostrocaudal organization to the frontal lobes, such that more abstract information is processed in anterior regions and more concrete information in posterior regions. Our data are well aligned with this account, as BA46, which is associated with color-blind performance, is anterior to BA 9, which is associated with sighted performance. The color-blind condition may be more abstract than the sighted condition, as it requires recognition of the inability of the avatar to appreciate color terms, a quality that is visually imperceptible and instead must be maintained in working memory. The sighted condition, on the other hand, is more concrete, with all cues explicitly available in the color, size, and pattern features of the competing objects. Unsurprisingly then, it is the sighted condition that shows a preferential overlap with the VVT (which similarly requires no abstraction beyond the objective appearance of the stimulus sets).

Also related to aberrant referential communication was the insula, which has been previously implicated in bvFTD (Seeley, 2010; Mandelli et al., 2016). Previous work has suggested that the insula is involved in guiding goal-directed behavior in dynamic social contexts and/or detecting salient events (Menon and Uddin, 2010; Bernhardt and Singer, 2012; Gasquoine, 2014). Thus, the insula may play a domain-general role in communication, helping to detect salient features (i.e., an interlocutor’s affect, gender, or social status) against a busy and constantly changing environment.

We also note here that the color-blind condition showed a unique relationship with inferior frontal gyrus pars triangularis (BA 45), one of the primary nodes of the classic language network. While this region is known to be active in cases of semantic ambiguity and when syntactic demands are high (Rodd et al., 2005; Hagoort and Indefrey, 2014), our two conditions (sighted, color blind) were perfectly matched in terms of semantic and syntactic load. Alternatively, our results seem to support previous work suggesting a role for the IFG in selection and interference resolution (Thompson-Schill et al., 2005; Nelson et al., 2009; Hagoort, 2014). Indeed, the color-blind condition is characterized by two opposing responses: both using the minimum number of requisite adjectives and identifying the target correctly, but one referring to color (to be rejected) and one referring to color or size (to be selected).

Finally, because diseases like FTD are thought to be network based (Seeley et al., 2009; Pievani et al., 2011), our last set of analyses examined the role that WM tracts may play in social perspective taking. Our analyses suggested that the uncinate fasciculus, which connects OFC to portions of the anterior temporal lobe and is broadly involved in social–emotional processing (Von Der Heide et al., 2013), may also be involved in referential communication. This result, which is consistent across sighted and color-blind conditions, as well as VVT, corresponds well to previous work showing that damage to the uncinate is predictive of bvFTD diagnosis (Agosta et al., 2012; Mahoney et al., 2014) and is associated with both impaired sarcasm identification (i.e., another example of language-based social coordination) and altered emotional empathy (Downey et al., 2015; Oishi et al., 2015). While early research on the language connectome focused primarily on the arcuate fasciculus (part of the superior longitudinal fasciculus connecting Broca’s and Wernicke’s Areas), the uncinate has also been included as part of the ventral stream in contemporary models (Dick et al., 2014), with some evidence demonstrating that it plays a role in semantic processing and naming (Agosta et al., 2010; Catani et al., 2013). According to our data, and given its physical architecture (i.e., the regions it traverses and connects), the uncinate likely plays a key role in daily communication by facilitating cross talk between the social and language networks.

### Caveats and future directions

While the findings described here are robust, several caveats should be kept in mind when interpreting our results. Although we were able to test a relatively large group of rare patients, our cohort was not pathologically confirmed and we did not have a brain-damaged control group, both of which would improve the specificity of our results. fMRI and/or repetitive transcranial magnetic stimulation studies in healthy adults using the same stimulus materials could offer converging evidence for our findings from an independent source. Next, while we are able to contribute to the ongoing debate regarding the relationship between executive function and social cognition in bvFTD, we did not examine comparative performance in younger adults, which would have helped us form a comprehensive model of referential communication in both healthy aging and disease. Similarly, we are unable to comment on the potential effects of gender or individual differences in attention. Finally, although previous work has demonstrated that language processing is comparable when interacting with a human-like avatar compared with a human partner (Heyselaar et al., 2017), the paradigm we developed used an avatar to represent a conversational partner. Future work might have greater ecological validity with truly interactive exchanges involving two human partners.

With these caveats in mind, the results of the present study support the view that impaired social coordination abilities in bvFTD are clearly evident in a novel referential communication task that carefully minimizes external task demands. Furthermore, the observed communicative impairment is due in part to limitations in mental set shifting and involves degradation of a prefrontal gray and white matter network that extends beyond the traditional, left peri-Sylvian language network.

## Synthesis

Reviewing Editor: Bradley Postle, University of Wisconsin Decisions are customarily a result of the Reviewing Editor and the peer reviewers coming together and discussing their recommendations until a consensus is reached. When revisions are invited, a fact-based synthesis statement explaining their decision and outlining what is needed to prepare a revision will be listed below. The following reviewer(s) agreed to reveal their identity: Katherine Rankin.

Reviewer #1

In this study, the authors investigated whether a group of patients with behavioral variant frontotemporal dementia (bvFTD) was impaired on a “social coordination” task. Specifically, the authors examined whether executive dysfunctions (set-shifting, inhibition, working memory) and changes in gray and white matter were associated with impaired social coordination in this patient sample. The results showed that impaired social coordination was associated with diminished mental set-shifting as well as changes in frontotemporal brain regions underlying executive functions and social cognition, including gray matter volume (orbitofrontal cortex, dorsolateral prefrontal cortex, insula) and white matter integrity (uncinate fasciculus).

Major comments:

• One of my main concerns is the experimental paradigm the authors used, which requires predominantly language and visuospatial functions and does not seem to be a good paradigm to measure “social coordination”. The patients' performance did not differ between the two conditions (sighted, colorblind), which calls into question whether the colorblind condition involves social cognitive processes (perspective taking). In addition, the visual-verbal task (set-shifting, executive function task) was associated with more social function regions than the colorblind condition, including the medial prefrontal cortex, which is not what one would expect if the colorblind condition required perspective taking abilities.

• The authors state that they included a non-aphasic bvFTD group but they do not provide any scores of language testing such as the BNT to confirm this. The atrophy map of the bvFTD group shows that the left temporal lobe was the region that was most affected, which highly suggests that at least some of the patients had semantic deficits that may have influenced their performance on the task.

• A related issue is that the authors calculated a correlation between the overall performance and the language composite score and concluded that the impairment they found in the bvFTD patients is independent from language. A better way to control for language functioning would be to perform general linear models and include language scores as covariates of no interest and see if the effect of group remains significant. This would also be important for the analysis in which the authors divided the patients into high and low performers. The differences between the groups could be due to differences in language functioning.

• The authors looked at both the gray and white matter correlates of social coordination, but they did not state any hypotheses with regard to which regions/tracts they expected to be associated with social coordination. Did they expect to see a relationship between social coordination and both imaging modalities?

• The authors used the NPI-disinhibition item as one of their executive function measures. However, the NPI-disinhibition item assesses social disinhibition but not cognitive disinhibition. The appropriate test to measure cognitive inhibition and thus executive functioning would be the Stroop interference condition test. The authors found that the NPI-disinhibition score was not related to the overall performance, which is likely because they used a measure of social disinhibition rather than cognitive disinhibition.

• The authors wrote that “The integrity of the WM tracts that is associated with the GM regions was identified by the patient' group's regression on colorblind performance”. It is unclear why the authors used this approach. Why did the authors only look at white matter tracts that were associated with gray matter atrophy of patients? Why didn't they look at white matter tracts based on a priori hypothesis of tracts that may be involved in social coordination?

• The authors found an association between white matter integrity in the left uncinate fasciculus and the overall task performance and conclude: “Patient impairment was related to disease in a network of prefrontal regions associated with social functioning and mental setshifting, highlighting the essential contribution of non-language brain regions to daily communication.” The left uncinate fasciculus has been shown to be mainly involved in language and not social functioning.

Minor comments:

• Methods, participants section: “Close inspection of individual patient profiles from our behavioral data suggested two subgroups within our patient population”. What do the authors mean by close inspection?

• The authors state that it was unexpected that they did not find a relationship with the medial prefrontal cortex, but they do not provide an explanation for this negative result. The medial prefrontal cortex is one of the core regions involved in perspective taking, which again calls into question if the paradigm used measures social aspects.

Reviewer #2

eN-NWR-0488-18

This study aims to establish the neuro-anatomical correlates underlying shared mental representations in communication (referred to as social coordination), by comparing performance of neurodegenerative patients on an adjusted referential communication task and neuropsychological tasks with gray matter atrophy and white matter integrity. It concludes that neurodegenerative patients do not sufficiently adapt communication behavior to their communication partner's perspective, which was connected with set-shifting difficulties in executive functioning, gray matter atrophy patterns in frontal and anterio-temporal regions, and left uniculate fasciculus tract changes. The study is a resourceful effort to fill in the gaps between the field of neurolinguistics and social neurosciences, and the found results support the current literature in these fields in a significant way. Noticeably, it reflects a strong ambition to identify all the processes that underlie language based social interactions. This however resulted in performance of many analyses, and some do not beneficially contribute to the hypothesis driven goal of the study, which makes interpretation of the results difficult. This study is actually a direct follow-up of a previously published analysis by this group of the gray matter correlates of this task, thus many of the previous results and methods are an important foundation for understanding this paper. However, many of these key points have not been clearly addressed here. A clear explanation for how this study extends and relates to their previously published work, and a more structured outline of the methods with a comprehensive explanation of the rationale behind the methods chosen here (which are slightly altered from the previous paper) as well as how these results relate to previously found results, would substantially contribute to the comprehensibility of the study. In general, clearer and more comprehensive explanations of key details throughout would significantly improve the manuscript.

MAJOR ISSUES:

Drawing a clear line from their previous work/better explaining rationale for study:

This study overlaps to a large extent with previously published work. Some alterations to the communication tasks are made, and additional neuropsychological and WM data is collected. Most novel finding of the present study is the correlation between EF (set shifting) and communication task performance, but it's important to state what results have already been published, and how the present study extends (or conflicts with?) these results. The following are some key omissions:

1) Introduction fourth paragraph: explain why bvFTD patients may be overwhelmed in generating continuous speech, are these results from previous work? It is necessary to explain this since simplifying the communication task takes away from the language aspect of the task, which is one of the objectives of the study.

2) There needs to be a better explanation for the reason why they conduct both GM and WM analyses (explain how it contributes to our general understanding of social coordination) and how these results relate to previous GM study. Why would white matter analysis be called for here? Consider extending this rationale to the hypothesis.

3) Third paragraph: When introducing the disease group, emphasize the rationale for why this group is included (e.g. from a lesion-model perspective or for clinical purposes, disease characteristics that fit the topic of interest). Connect this with the literature that is cited.

4) Methods experimental design: Is there any proof that the referential communication task really is testing perspective taking (difference between sighted vs colorblind)? This evidence should be stated clearly.

5) Discussion: an explanation of the discrepancy in the behavioral results that were found in PT is missing. bvFTD patients use superfluous language that would indicate problems in PT, but when results from sighted and colorblind subjects are compared with each other, bvFTD patients do not have significantly more difficulties with the perspective taking item (colorblind).

Also, results related to the mPFC are indicating that the referential communication task might not explicitly measure PT. I would either find a way to overturn this argument, or acknowledge.

MINOR ISSUES/DETAIL:

Introduction

First paragraph: Relevance of perspective taking on generating a counter-response; I would recommend breaking down what perspective taking entails (i.e. what processes are involved in perspective taking). From there you can argue why EF is important for social coordination.

The relevance of healthy social coordination might be more explicitly explained. Pointing out that it influences relationship quality and life satisfaction requires more background information (or a reference to another paper at minimum).

Second paragraph: mentioning multimodal imaging requires an example of a multimodal method study. The examples given in this paragraph appear to only be unimodal.

Fourth paragraph: What are the considerations to include EF? Need to explicitly connect EF problems with aberrant social communication. Recommend to describing why the Miyake EF model was adopted.

Methods

Participants section: sample, not population. There is no explanation for why gender was not included as a potential confound in any of their analyses; this seems like it might be an important factor for social communication, proof should be provided that it is not if it is going to be excluded thereafter.

Neuropsychological battery section: state why language measures were included in the methods (and why these specific measures).

Experimental design section: Explain how the adjusted referential communication task is scored. Related: Explain in figure 1 description that participants were asked to select the right answer based on a minimal number of descriptors.

Explicitly point out that the number of competitors variables aim to test working memory capacities (instead of ‘recourse demands’)

Star in figure; because both the superfluous and correct answer are used in analyses, adding a star only to the correct answer is a little suggestive, perhaps more explanation for this choice would make it clearer.

Structural imaging section: include demographic information on the 18 bvFTD patients (2 less than described in participant section), or point out (if that is the case) that no sig. differences exist between this group and the total group of 20 patients.

Explain more clearly why a second permutation analysis was conducted.

Typo: chance of type 2 error, not change.

DTI section: Perhaps move explanation of WM influence on behavior to the introduction section to better set up rationale.. Explain why in WM integrity analysis only the colorblind group was analyzed.

Results

Behavioral results section:

Influences of age, disease severity, language and visuoperception are controlled for, but influence of gender and attentional processes are not controlled. Considering that bvFTD patients are known to have problems in attention processing, stating that patients' impairment can only be due to social or EF impairments is premature. The absence of an attentional ‘task control’ test should be mentioned in the limitations section.

Difference between the sighted and colorblind conditions; I would specifically state that this condition tests perspective taking (to remind the reader), and the effect of higher response accuracy in healthy controls can be due to statistical differences between the two formats, where the colorblind condition basically adds another clue to which answer is the right answer, increasing changes of correctly answering the question. Absence of this effect in bvFTD patients shows that this group tends to respond in a superfluous way.

Effect of competitor number; patients tend to give superfluous answers (independent of competitor number), which could also explain the absence of a working memory effect. Taking response style as a variable into the model as a confound could control for this. Combined with the NPI result, this would strengthen the argument that working memory does not play a role in social coordination.

Creating a separate subtitle for neuropsychological testing makes the reader think that these are used as control measures, whereas they are actually used for interpretation of the effects in the discussion; keep under behavioral result section or create more subtitles.

Imaging results section: emphasize in results (or methods) that interpretation of patient vs. control GM atrophy results are reflected as a disease control because the pathological confirmation of diagnosis is missing. This helps the reader understand why this analysis is conducted (otherwise rather unnecessary).

Explain what the purpose is of comparing visual-verbal test related GM atrophy and colorblind related GM atrophy. This is not described in the methods which makes it difficult to follow.

Subgroup analyses in patients: recommend to add behavioral analyses to the behavioral section (earlier in the results). Also recommend not to refer to these analyses as individual patient analyses since this still represents 2 patient groups (selected based on individual characteristics).

Add indication of left and right hemisphere to images.

Tractography section: expected results reflected in last part method section (1) associated GM regions, 2) changes in patients, 3) associated performance) are not explained.

Comparison with overall task performance is actually comparison with the colorblind performance. One could assume that overall task performance refers to how many errors were made in the whole task, both colorblind and not colorblind.

I also wonder why WM integrity is not compared with the EF set-shifting test (being consistent with conducted analyses)

Discussion

Statement that findings contribute to daily conversation is slightly misplaced since the task is not highly ecologically valid (as mentioned in limitation section); This again shows it is necessary to explain how results should be understood on a real-life naturalistic level.

As mentioned in result section, difference between patients and healthy controls could be caused by higher likelihood chances in the test.

Multi modal network section: give an example of salient features.

Network based effects do also have an effect on GM intensities, important to further explain the relation (and meaning of results) between the GM and WM results.

Limitations: explain how healthy adult data would add additional support.
